# MyotonPro Is a Valid Device for Assessing Wrist Biomechanical Stiffness in Healthy Young Adults

**DOI:** 10.3389/fspor.2022.797975

**Published:** 2022-02-21

**Authors:** Anh Phong Nguyen, Christine Detrembleur, Paul Fisette, Clara Selves, Philippe Mahaudens

**Affiliations:** ^1^Neuromusculoskeletal Laboratory, Institut de Recherche Expérimentale et Clinique, Catholic University of Louvain, Louvain-la-Neuve, Belgium; ^2^Institute of Mechanics, Materials and Civil Engineering, Mechatronic, Electrical Energy and Dynamic Systems, Catholic University of Louvain, Louvain-la-Neuve, Belgium; ^3^Service de Médecine Physique et Réadaptation, Cliniques Universitaires Saint-Luc, Woluwe-Saint-Lambert, Belgium

**Keywords:** myotonometry, viscoelasticity, myofascial stiffness, elastic, viscous, musculoskeletal

## Abstract

**Background:**

The MyotonPro is a portable device for measuring biomechanical and viscoelastic properties in superficial soft tissues. The aims of this study are firstly to validate the MyotonPro compared to a reliable gold-standard frame and secondly to observe the influence of MyotonPro measurement on the total wrist viscoelasticity.

**Methods:**

Three silicone polymers with different elastic properties were assessed with the MyotonPro and with a reference rheometer (Universal Tribometer Mod). Then, a free oscillations method was used to measure the passive elastic and viscous stiffness of the wrist and compared to MyotonPro forearm measurements.

**Results:**

A one-way ANOVA demonstrated the validity of the MyotonPro's stiffness (*p* = 0.001), decrement (*p* < 0.001), and relaxation (*p* = 0.008) parameters for measuring the elastic stiffness (*k*) of the three polymers. The MyotonPro parameters demonstrated excellent reliability on the forearm. Proximal and distal anterior myofascial measurements of the MyotonPro were moderately correlated to the elastic stiffness (*p* = 0.0027–0.0275, absolute *r* = from 0.270 to 0.375) of the wrist while the postero-distal myofascial tissues of the forearm demonstrated a moderate correlation with the viscous stiffness of the wrist (*p* = 0.0096–0.0433, absolute *r* = from 0.257 to 0.326).

**Discussion:**

The MyotonPro is a valid device for measuring elastic stiffness as well as a portable, affordable, and easy-to-use tool for quantifying the biomechanical properties and viscoelasticity of myofascial tissue in healthy subjects.

## Introduction

The understanding of the biomechanical and viscoelastic stiffness of the musculoskeletal system is of great interest in numerous fields such as health, sports, and physical activities. These properties are important components of joint stability and movement control (Stanev and Moustakas, 2019). In mechanical engineering, this stiffness, or *k*, can be defined as the relationship between the applied stress and the induced strain in a system/structure. In biomechanics, we use the term “elastic stiffness” to define the “*k*” (Butler et al., 2003). This term differs from “elasticity,” which is used sometimes and refers to the damping effect of a tissue, e.g., skin elasticity. The passive stiffness is thought to be induced by myofascial tissue such as the muscle, the fascia or the tendon. Furthermore, the joint capsule and ligaments are also involved in overall stiffness (Butler et al., 2003). In health, the quantification of stiffness is relevant in neurological disorders, e.g., spasticity (Detrembleur and Plaghki, 2000) and Parkinson's disease (Rätsep and Asser, 2011) or musculoskeletal injuries such as tendinopathy. It allows a better understanding of the pathophysiology of these disorders, as well as better follow-up and treatment decision-making. In sports, the assessment of stiffness allows a better understanding and thus, planification of physical performance and prevention of sport related health problems (Arampatzis et al., 1999; Brughelli and Cronin, 2008; Maloney et al., 2016).

The MyotonPro (Myoton AS, Tallinn, Estonia) is a non-invasive hand-held, affordable, and easy-to-use myotonometer device aimed at recording the biomechanical and viscoelastic stiffness of myofascial tissues (Bizzini and Mannion, 2003; Aird et al., 2012; Schneider et al., 2015; Zinder and Padua, 2017). The data obtained with the MyotonPro consist of five parameters. Three of them correspond to biomechanical stiffness: frequency, representing the tonus or state of active tension, dynamic stiffness and decrement (or elasticity). The last two represent viscoelastic stiffness: relaxation time and creep (Schneider et al., 2015). Many studies have assessed its feasibility and reliability in a healthy population, both young and older individuals (Agyapong-Badu et al., 2013, 2016), in spastic patients (Lo et al., 2017; Drenth et al., 2018) and in musculoskeletal disorders (Jiménez-Sánchez et al., 2018). The MyotonPro elicits a good to excellent reliability in the quadriceps femoris (Dellalana et al., 2019), the biceps brachii (Deun et al., 2017), the upper trapezius (Kawczyński et al., 2018), the paraspinal muscles (Hu et al., 2018), the gastrocnemius and Achilles tendons (Taş et al., 2019), and the pelvic floor muscles (Davidson et al., 2017). Previous studies did not usually assess the validity of the five parameters together and focused only on one or two of interest, with stiffness as the main component. To the best of our knowledge, no study has determined the validity of the five parameters with respect to an accurate and valid gold-standard measurement tool for elastic stiffness (ES) or a device that differentiates elastic from viscous stiffness (VS).

The free oscillations technique is one of the valid methodologies used to quantify viscoelasticity in the extremities of the joints of limbs (Detrembleur and Plaghki, 2000; Nguyen et al., 2020). The electromechanical oscillation device (EOD) is a proven, reliable tool for assessing and quantifying the passive elastic and viscous stiffness of the ankle (Lobet et al., 2018) or wrist joint (Nguyen et al., 2020). Elastic and viscous stiffness represent respectively the spring-like and damper-like properties of tissues. However, while the EOD can measure the total stiffness of a joint, it cannot identify the involvement of the capsule joint, ligaments, muscle, or tendon tissues in the total measured stiffness.

The aims of the present study are: (i) to assess the validity of the five parameters measured by the MyotonPro compared to an accurate and reliable testing machine tool, a tribometer, which assesses biomechanical properties using silicone polymers; and (ii) to establish the relationship between forearm myofascial tissue, as measured by the MyotonPro, and its correlation to ES and VS of the wrist in healthy young adults, as measured by the EOD.

## Methods

This observational study was performed by using two standard reference systems, a tribometer and the EOD. The accuracy of the five MyotonPro parameters in assessing ES was evaluated on three different polymer surfaces and was compared to the tribometer assessment. The relationship between the passive stiffness of the wrist as assessed by the EOD and the measured stiffness of posterior and anterior, distal and proximal myofascial tissues of the forearm as assessed by the MyotonPro, was evaluated on healthy young volunteers. This study received the approval of the local ethics committee (Comité d'Ethique Hospitalo-Facultaire) from the Université Catholique de Louvain (UCLouvain, Belgium—N°: B403201942384) and was performed in accordance with the principles of the Declaration of Helsinki. All participants provided written informed consent.

## Materials

### MyotonPro

The MyotonPro produces a series of damped oscillations induced by a single external mechanical impulse. The probe of the MyotonPro was placed perpendicularly to the assessed structure and, with a pre-force of 0.18 N, a quick mechanical impulse of 0.4 N was released for 15 ms. The total force applied to the structure was 0.58 N. Following the single external mechanical impulse, free oscillations were recorded for 400 ms. Then, the device needed 10 ms for processing raw signals and five more to compute all the parameters. The sequence was looped five times for a total duration of 2.15 s. The mean value of the five sequences corresponds to one assessment allowing the calculation of five parameters: frequency, stiffness, decrement, relaxation time, and creep. Frequency (F) corresponds to the oscillation frequency and represents either the tonus of the muscle or the state of tension if the muscle is contracted:


(1) $$\begin{array}{r} F\;\left[Hz\right]=Fmax \end{array}$$

**Figure 1:**
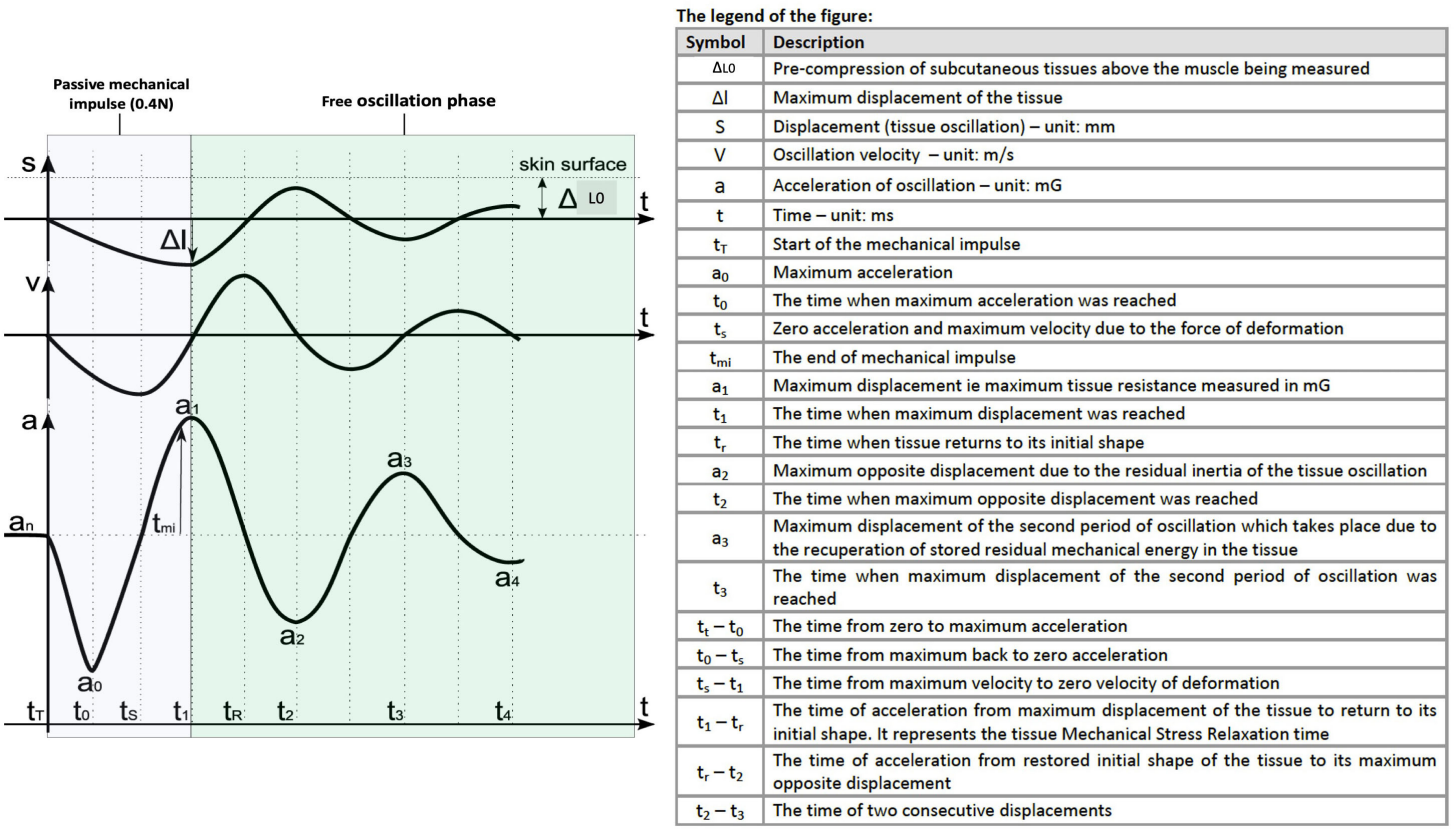
Description of the induced mechanical impulse and the free oscillation phase and the relationship with displacement (l), velocity (V), and acceleration (a). Adapted from the MyotonPro user manual (MyotonPro AS, London, United Kingdom) and previously described by Schneider et al. (2015).

Stiffness (S) should represent the k of the structure:


(2) $$\begin{array}{r} S\;\left[\frac{N}{m}\right]=\frac{a_{\max}\;.\;m}{l} \end{array}$$

where a = acceleration of the probe, m = the mass of the probe and *l* = displacement. The decrement (D) is described as the logarithmic decrement of acceleration between the first and second cycle. It represents the loss of energy and is described as the elasticity of the system:


(3) $$\begin{array}{r} D=\ln\;\left(\frac{a_{1}}{a_{3}}\right) \end{array}$$

where a_1_ and a_3_ are the maximal accelerations of the first and second cycles, respectively. A decrement value of 0 would represent a perfect elasticity with no loss of elastic energy (Figure 1). Relaxation time (R) is the delay between the maximal value and the end of the first cycle. It represents the time that the structure needs to recover its initial position. Creep (C) is a representation of the Deborah number. It is a dimensionless number that characterizes the fluidity of a material under specific conditions (Poole, 2012). As regards to the MyotonPro methodology, it is defined as the gradual elongation of tissue over time when placed under a constant tensile stress (Schneider et al., 2015). The more solid-like the material is, the higher the creep value will be.

### Tribometer and Polymers

**Figure 2:**
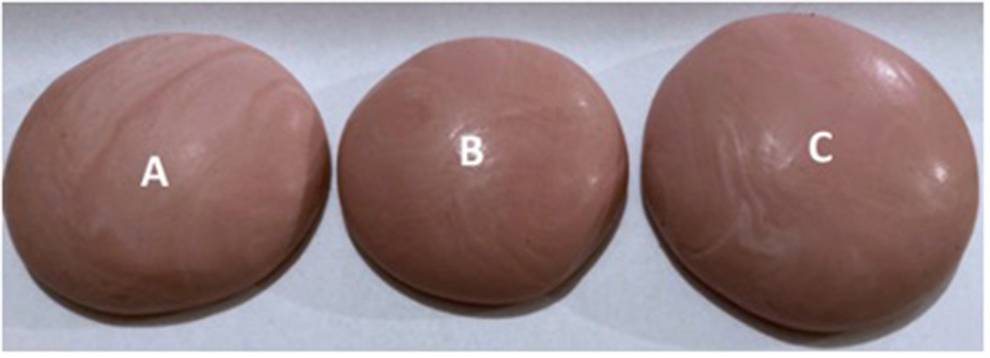
Presentation of the tested polymers. The polymers were shaped free on a plane surface. Diameter/height (in cm): **(A)** 5.5/1.7, **(B)** 5.1/1.6, **(C)** 5.6/1.7.

The ES of the polymers was assessed using a universal tribometer mod (UMT-3MT) equipped with a testing block, s/n T45185 (Bruker-Nano Surfaces Division, Campbell, CA, USA). Polymers (Figure 2) were created using “Poly-addition” silicone SICASIL materials (Capron Podologie, Ecuisses, France). Different dosages of catalysis and base layers (1:1, 1:2, 2:1) were adjusted to obtain three polymers of different density and stiffness. The tribometer device executed two tests at three different velocities (0.5, 1, and 2 mm s^−1^) to verify the k, which was independent of speed, for each polymer. The *k* of each polymer was calculated as the slope of the vertical force (Fz recorded during each tribometer test at 1,024 Hz) over the vertical displacement (simultaneously recorded). All measurements were taken in the middle of the top of the polymers, which was marked with an indelible dot in order to standardize all measurements at the same testing spot. MyotonPro measurements were assessed twice on each polymer and compared to the *k* of each polymer.

### Electromechanical Oscillation Device (EOD) in Healthy Subjects

**Figure 3:**
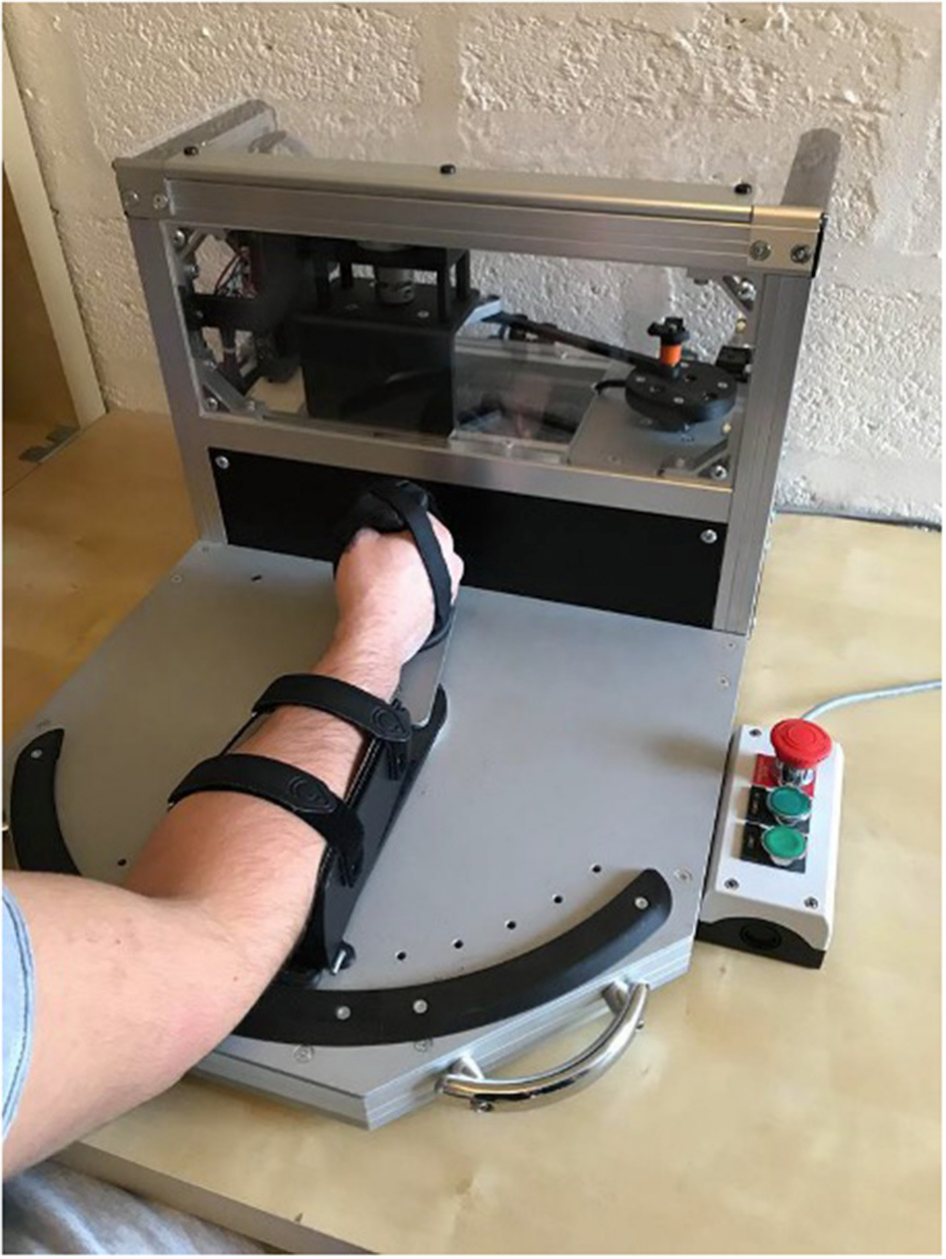
Position of the wrist into the electromechanical oscillation device (EOD).

The EOD generated sinusoidal rotatory movements to assess the viscoelasticity of the wrist (Rack, 1966; Lehman and Calhoun, 1990; Detrembleur and Plaghki, 2000). The EOD induces a passive flexion/extension movement (±10°) of the wrist at several frequencies from 3 to 12 Hz. A potentiometer and a torque meter record the angular displacement and torque. By computing the data and assuming that the subject does not exert any voluntary contraction, the EOD extracts the ES and VS values of the wrist. Elastic stiffness is defined as the restoring force that acts like a spring mechanism and is directly proportional to the deformation of the structure and independent of the velocity of the deformation, as opposed to VS, which acts as a damper and which is correlated to the velocity of the deformation. EOD technology has been validated as a safe, reliable device for measuring passive viscoelastic stiffness in healthy subjects (Nguyen et al., 2020). The participants sat in front of the EOD, with their elbow flexed at about 120° and their forearm relaxed. The wrist was firmly attached to the gripping handle to keep the fingers passively closed around the handle. The fingers are held in this way thanks to a strap in the form of a glove enclosing the fingers around the handle with a velcro system to prevent the fingers from opening and releasing the handle (Figure 3). Only the dominant wrist was tested. The participant either began with the EOD or the MyotonPro measurement, following a simple randomization (with sealed letter).

**Figure 4:**
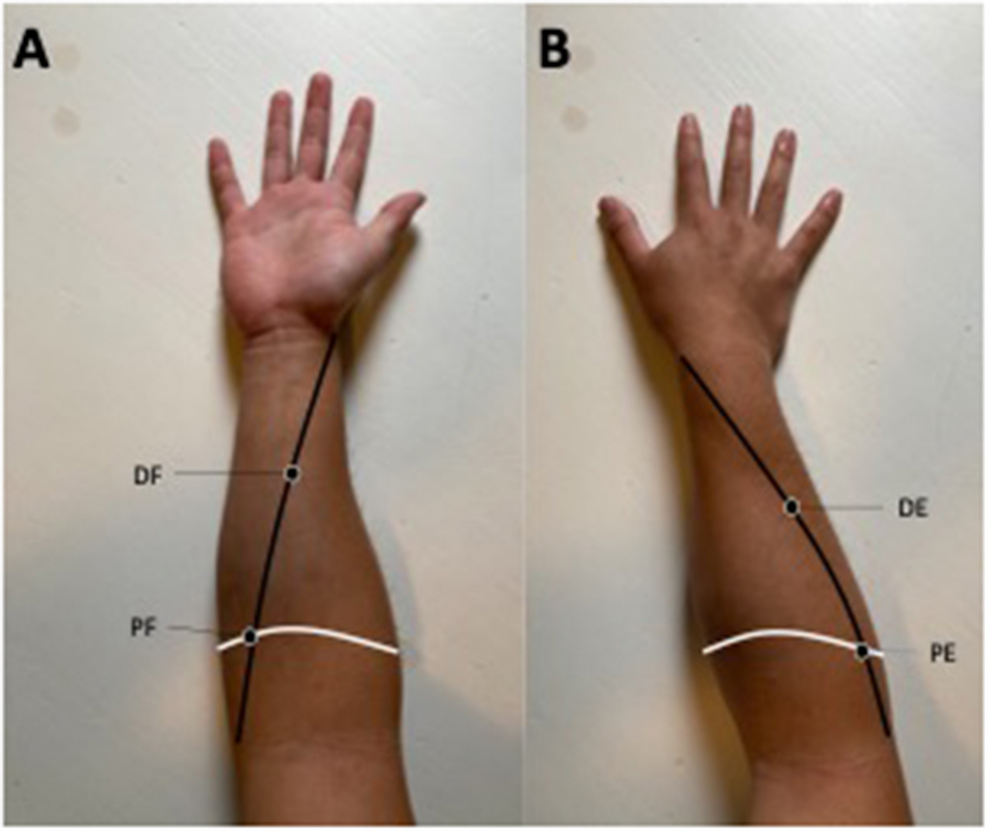
**(A)** Represents the anterior face of the forearm while **(B)** represents the posterior part of the forearm. The white line determines the most prominent part of the forearm. The black line represents the line from the medial epicondyle **(A)** and the lateral epicondyle **(B)** to the radial styloid. These lines determine the four measurement sites: PF, the proximal flexor; DF, the distal flexor; PE, the proximal extensor; DE, the distal extensor. This figure represents the position of point of measurement and not the position of the MyotonPro protocol measurement.

The MyotonPro was applied at four different points of the dominant forearm. Two lines were drawn from both medial and lateral epicondyles of the humerus to the radial styloid to delimit the four measurement sites. The intersection of these lines at the most prominent part of the forearm represented the first site, on the palmar side, the proximal flexor (PF), and on the dorsal side, the second site, the proximal extensor (PE). The middle of the line between the PF and the radial styloid and the PE and the radial styloid corresponded to the third and fourth sites, respectively the distal flexor (DF) and distal extensor (DE), see Figure 4. The hand of the patient was placed in neutral position using an external support with the finger relaxed in a flexed position.

### Participants

Concerning the reliability study, a sample of convenience of 30 healthy participants were assessed twice by the same investigator. Then the wrist stiffness was assessed using both the MyotonPro and the electromechanical oscillation device (EOD) in 52 healthy participants. The sample size is based on the calculation that a random sample of 44 subjects produces a two-sided 90% confidence interval with a width of 0.099 when the estimated intraclass correlation is 0.900. Participants were included if they were between 18 and 30 years old, presented no musculoskeletal disorders of the upper limb and had not engaged in any physical activity within the 24 h prior to the experience. Any participants that suffer from any musculoskeletal injuries or pain, neurological disorders or underwent any type of surgery on the upper limb were systematically excluded. The participants were recruited from the university and surroundings areas (UCLouvain, Belgium) and received no financial compensation.

### Statistical Analysis

Statistical analysis was performed using MedCalc Statistical Software version 16.4.3 (MedCalc Software, Ostend, Belgium).

#### Tribometer vs. MyotonPro: Polymer Surfaces

A One-Way ANOVA was performed in order to test the effect of three different polymer densities on *k* parameters (tribometer) and MyotonPro's parameters (F, S, D, R, and C). A *post-hoc* analysis was performed using the Holm-Sidak method. Measurements between the three polymers were also expressed in per cent. Furthermore, spearman correlation was performed in order to assess any correlation between Tribometer and MyotonPro values.

#### EOD vs. MyotonPro: Healthy Participants

The reliability assessment was executed with all the measurement location, i.e., PF, DF, PE, and DE. To determine the Intraclass correlation coefficient, a two-way mixed model with absolute agreement was performed. Furthermore, standard error of the mean (SEM) and minimal detectable change (MDC) were calculated. In order to compare EOD and MyotonPro measurements in healthy subjects, we normalized all parameters as the units of those parameters were different and could not be compared without standardization. More precisely, we processed all parameters following this equation:


(4) $$\begin{array}{r} normalized\;\left(x\right)=\frac{x-\min\left(x\right)}{\max\left(x\right)-\;\min\left(x\right)} \end{array}$$

where *x* represents the parameter of interest. We performed a Passing & Bablok linear regression (Bablok and Passing, 1985) on each normalized value for MyotonPro (X variables), i.e., F, S, D, R, and C, compared to EOD (Y variable), i.e., ES or VS. The intercept (A) of the linear regression were calculated with a 95% interval confidence. The intercept A is a measure of the systematic difference between the two methods. This hypothesis is accepted if the confidence interval for A contains the value 0. As the Intercept (A) represents the systematic difference, Bland-Altman plots was not performed. As data were not normally distribute (Shapiro-Wilk test), a Spearman rank correlation coefficient was calculated for all significant interactions. The level of significance for all statistical analysis was *p* < 0.05.

## Results

### Tribometer vs. MyotonPro: Polymers

Tribometer measurements demonstrated significant differences between the three polymers (*p* < 0.001) with polymer B = 1.436 ± 0.024, C = 2.42 ± 0.036, and A = 2.732 ± 0.021 Nm^−1^. MyotonPro statistical results showed significant differences between polymers for stiffness (*p* = 0.001, B = 1341.0 ± 25.45, C = 1494 ± 5.65, and A = 1610.5 ± 5.65 Nm^−1^), the decrement (*p* < 0.001, B = 0.35 ± 0.01, C = 0.41 ± 0.001, and A = 0.61 ± 0.007) and relaxation time (*p* = 0.008, B = 6.05 ± 0.07 s, C = 5.8 ± 0.001 s, and A = 5.55 ± 0.07 s). For those three parameters, *post-hoc* analysis (holm-sidak) showed that all parameters were different between all polymers. Frequency (B = 60.8 ± 6.22, C = 56.9 ± 0.77, and A = 65.2 ± 5.44 HZ) and Creep (B = 0.65 ± 0.01, C = 0.62 ± 0.001, and A = 0.61 ± 0.01) did not demonstrate any significant difference between polymers.

**Figure 5:**
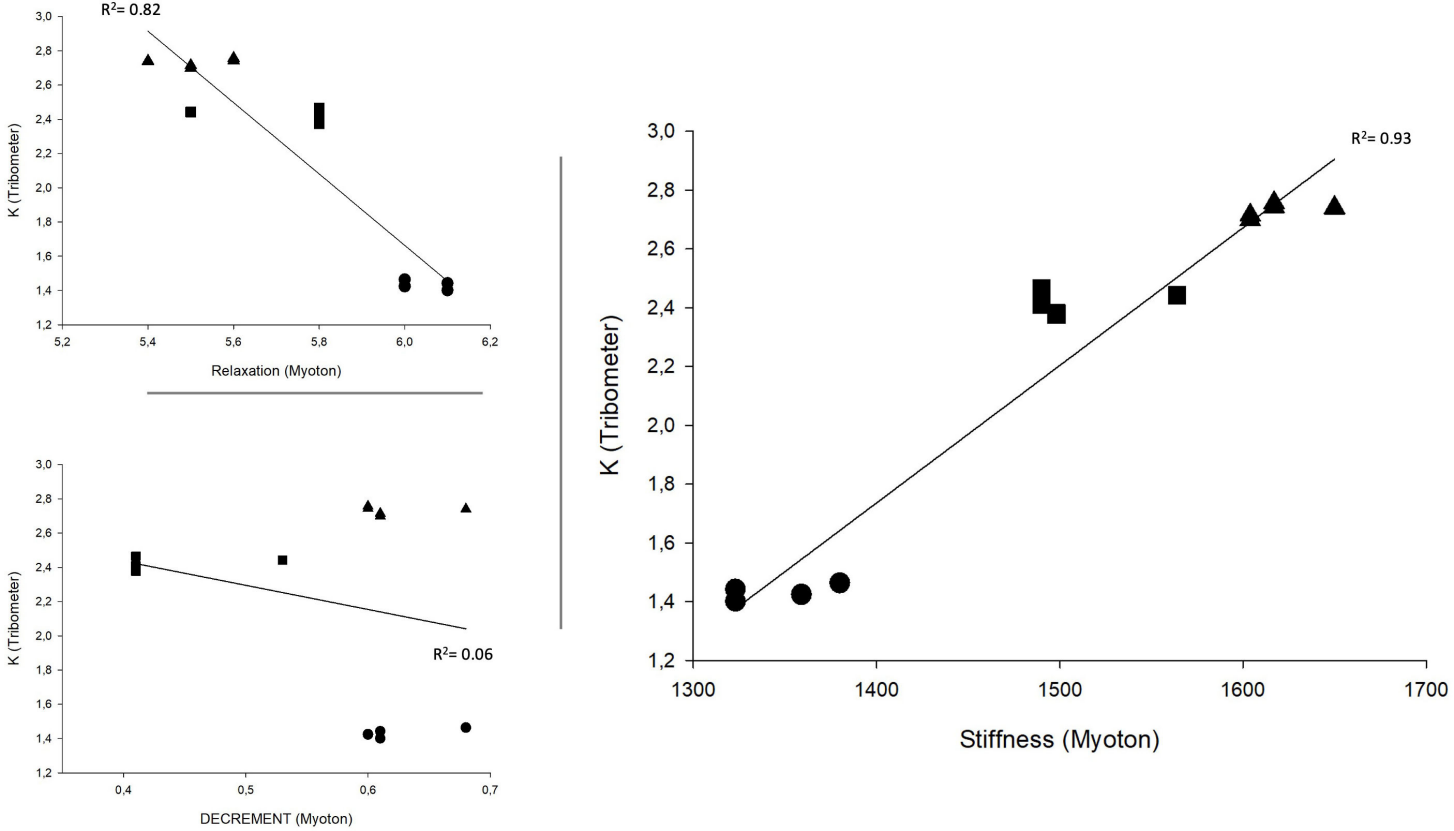
Correlation between Tribometer and MyotonPro values. Triangle represents polymer A, circle represents polymer B, and square represents polymer C.

The change assessed by the tribometer and expressed in per cent showed a 47% difference between Polymers A and B, 11% between Polymers A and C and 36% between Polymers B and C. Similar changes were observed with the MyotonPro for the stiffness parameters, respectively 42, 17, and 32%. The changes observed between polymers for D and R were not comparable (17, 7, and 11% for D; 9, 5, and 4% for R) (Figure 5).

### EOD vs. MyotonPro: Healthy Participants

The reliability assessment was performed on 30 healthy participants (17 men and 13 women: 22.1 ± 2.76 years, 177.7 ± 7.35 cm, 73.7 ± 12.29 kg). The ICC were excellent for all parameters with the exception of decrement, who demonstrated moderate reliability (Table 1).

**Table 1:** Intraclass correlation coefficient of MyotonPro measurements on the forearm.

	**ICC**	**95% CI**	**SEM**	**MDC**
Frequency (Hz)	0.93	0.92–0.94	0.66	1.83
Stiffness (N/m)	0.95	0.94–0.96	19.04	52.77
Decrement	0.83	0.79–0.85	0.102	0.29
Relaxation (s)	0.95	0.94–0.96	0.77	2.14
Creep	0.94	0.93–0.05	0.05	0.13

*ICC, intraclass correlation coefficient; 95% CI, 95% confidence interval; SEM, standard error of the mean; MDC, minimal detectable change. All ICC comprised the MyotonPro measurement of the proximal and distal flexor (PF, DF) and proximal and distal extensor (PE, DE)*.

Of the 52 participants, all were included in the study. There were 31 men and 21 women (22.8 ± 2.37 years, 173.8 ± 9.42 cm, 69.9 ± 11.68 kg). With the exception of the decrement of the DF, PE, and DE, no systematic differences were found between the MyotonPro's parameters and ES values (Table 2). Likewise, no systematic differences were found between MyotonPro and VS values, except for the decrement of the PE. Due to the normalization of values, no proportional differences were identified. Spearman rank correlation coefficients can be seen in Table 2. The MyotonPro's parameters such as frequency, stiffness, relaxation, and creep in the flexor sites were significantly (*p* < 0.05) correlated to ES. As for VS, only the stiffness and relaxation in the PF were significantly correlated (*p* = 0.03 and *p* = 0.04). Finally, the frequency, stiffness, relaxation, and creep of the DE were significantly (*p* < 0.05) correlated to VS.

**Table 2:** Results of Passing and Bablok linear regression.

**EL**	**Systematic differences**		**Spearman rank correlation**	
	**Intercept A**	**95% CI**	***p*-value**	***r***
PF-Frequency	0.04144	−0.1120 to 0.1479	**0.0279**	**0.279**
PF-Stiffness	−0.09245	−0.2438 to 0.03488	**0.0102**	**0.324**
PF-Decrement	0.1732	−0.08000 to 0.3308	0.1821	
PF-Relaxation	0.04439	−0.4201 to 0.2746	**0.0077**	**−0.336**
PF-Creep	0.02448	−0.4306 to 0.2095	**0.0149**	**−0.308**
DF-Frequency	0.146	−0.01771 to 0.2205	**0.0063**	**0.344**
DF-Stiffness	0.06226	−0.08075 to 0.1996	**0.0092**	**0.328**
DF-Decrement	0.2014	0.003487 to 0.3358	0.7672	
DF-Relaxation	0.1798	−0.1835 to 0.3580	**0.0027**	**−0.375**
DF-Creep	0.1625	−0.2249 to 0.3466	**0.003**	**−0.371**
PE-Frequency	−0.01575	−0.3068 to 0.1398	0.6302	
PE-Stiffness	−0.2106	−0.7430 to 0.09951	0.3372	
PE-Decrement	0.4113	0.2125 to 0.5311	0.0126	−0.315
PE-Relaxation	−0.06436	−0.3771 to 0.05531	0.3671	
PE-Creep	−0.03836	−0.3277 to 0.08602	0.3376	
DE-Frequency	0.1276	−0.1456 to 0.2620	0.3618	-
DE-Stiffness	0.1441	−0.01739 to 0.2392	0.3933	-
DE-Decrement	0.2688	0.1519 to 0.3655	0.8229	-
DE-Relaxation	0.1777	−0.01753 to 0.2694	0.1834	-
DE-Creep	0.1678	−0.03159 to 0.2813	0.1559	-
**VI**	**Systematic differences**		**Spearman rank correlation**	
	**Intercept A**	**95% CI**	***p*** **-value**	***r***
PF-Frequency	0.002473	−0.2905 to 0.1918	0.1408	
PF-Stiffness	−0.1217	−0.3473 to 0.04591	**0.0354**	**0.268**
PF-Decrement	0.1585	−0.1376 to 0.2980	0.3276	
PF-Relaxation	0.09816	−0.4260 to 0.2636	**0.0433**	**−0.257**
PF-Creep	0.047	−0.5253 to 0.2239	0.0689	
DF-Frequency	0.1122	−0.06703 to 0.2658	0.4079	-
DF-Stiffness	−0.009202	−0.2599 to 0.2354	0.5557	-
DF-Decrement	0.1914	−0.03304 to 0.3133	0.6392	-
DF-Relaxation	0.06471	−0.2544 to 0.2210	0.3085	-
DF-Creep	0.04279	−0.2409 to 0.2084	0.336	-
PE-Frequency	−0.1727	−0.7130 to 0.06490	0.2951	-
PE-Stiffness	−0.4413	−1.9636 to 0.00462	0.0651	**-**
PE-Decrement	0.401	0.08415 to 0.4984	0.1304	-
PE-Relaxation	−0.1517	−0.4068 to 0.02574	0.0807	-
PE-Creep	−0.1258	−0.3590 to 0.05175	0.0748	-
DE-Frequency	0.04843	−0.3547 to 0.2464	**0.0254**	**−0.284**
DE-Stiffness	0.1513	−0.1095 to 0.3045	**0.0141**	**−0.31**
DE-Decrement	0.1673	−0.04759 to 0.3092	0.8714	–
DE-Relaxation	0.1433	−0.02971 to 0.2612	**0.0097**	**0.326**
DE-Creep	0.1487	−0.05152 to 0.2684	**0.0096**	**0.326**

*No systematic difference was established if the 95% CI (confidence interval) was 0. Significant Spearman correlation p-values are indicated in bold*.

*r, coefficient correlation (in bold); PF, proximal flexor; DF, distal flexor; PE, proximal extensor; DE, distal extensor*.

*MyotonPro parameters correlated to the EOD measurements are indicated in bold and underlined*.

## Discussion

The first aim of this study was to assess the validity of the five MyotonPro parameters in regard to the ES. We used silicone polymers with different stiffness and compared the gold standard, i.e., the tribometer, with the MyotonPro measurements. We found that *k* could be validly assessed by the stiffness parameter. Secondly, we demonstrated an excellent reliability to all MyotonPro parameters with the exception of D who demonstrated a moderate reliability. Finally, we assessed the influence of the biomechanical and viscoelastic properties of the forearm myofascial tissues measured by the MyotonPro on the passive stiffness of the wrist joint tested with the EOD. We observed a small correlation between the anterior forearm tissue for F, S, R, and C and the ES of the wrist. Also, a small correlation between the distal posterior tissue and the proximal and anterior tissue of the forearm F, S, R, and C was found in regard to the VS.

### Tribometer and Polymers

To the best of our knowledge, this is the first study that compared the five MyotonPro parameters to an accurate and valid device to measure polymer elasticity. Sohirad et al. (2017) previously used gelatin polymers and compared piezoelectric accelerometer measurements to MyotonPro measurements. They found that stiffness and decrement were consistent with the modification of gelatin concentration (and therefore the stiffness, *k*). Another study found that stiffness measured with the MyotonPro was highly correlated (*r*^2^ = 0.96) with Young's modulus of a gel-based polymer, calculated with a compression test of 100 N at a velocity of 20 mm/min (Dougherty et al., 2013). Based on our results, we confirmed that stiffness and decrement parameters are related to elastic stiffness, ES or *k* measurements. The stiffer a structure is, the more acceleration it will induce on the MyotonPro's probe, increasing the stiffness parameter. Likewise, the less stiff it is, the more energy it will release between two oscillation peaks, decreasing the decrement value, and thus increasing the elasticity. We also observed that relaxation time was related to elastic stiffness. Conversely, we could not confirm that frequency and creep parameters were relevant in assessing passive elastic stiffness. This might be due to a lack of validity among those parameters.

Furthermore, an *in vivo* study compared the MyotonPro parameters to ultrasound shear wave elastography in myofascial tissue (Kelly et al., 2018). Lohr et al. (2018) compared MyotonPro parameters with tensiomyography measurements. Both studies demonstrated that MyotonPro parameters were more sensitive to modifications of joint position (Liu et al., 2018; Lohr et al., 2018) or intensity of muscle contraction (Kelly et al., 2018). We confirmed that the MyotonPro demonstrated sufficient accuracy in detecting small changes in elastic-related stiffness.

### EOD and Healthy Participants

This is the first study to investigate the local biomechanical and viscoelastic properties of the forearm myofascial tissue and its relationship with the passive stiffness of the wrist. It is well-known that the passive stiffness of the wrist is multifactorial (Riemann, 2012). The wrist's passive stiffness could be induced by several biological structures, such as the muscle-tendon unit, the skin, the subcutaneous tissue, the fascia or ligament, the cartilage, or the joint capsule (Riemann, 2012). Also, external factors could influence the passive stiffness, such as ambient or body temperature, blood flow, alcohol consumption, time of the assessment, or physical activities (Deun et al., 2017). A device such as the EOD can determine the ES and VS of the wrist joint but cannot differentiate the influence of the biological structures surrounding the wrist region (Nguyen et al., 2020).

Zonnino and Sergi (2019) demonstrated the influence of the fingers and thumb muscles in the passive stiffness of the wrist with a computational framework based on a musculoskeletal model in OpenSim. We partially confirmed this hypothesis with our results. The anterior myofascial tissues of the forearm showed a small but significant correlation with the ES of the wrist measured by the EOD in healthy subjects. Furthermore, our study was the first to compare MyotonPro parameters with viscous stiffness as assessed by the EOD. In the wrist, our results showed that the DE and stiffness and relaxation time of the PF were related to the viscosity of the wrist. In our opinion, the MyotonPro cannot measure all the surrounding tissues involved in wrist stiffness as several structures cannot be palpated. The MyotonPro is described as relevant if the tissue of interest is palpable and located at least 2 cm below the skin. In contrast, the decrement value, representing elasticity or the energy loss following the mechanical impulse, did not demonstrate any significant correlation with either ES nor VS. The stiffness parameters seemed to be the most relevant parameters measured with the MyotonPro. In addition, Pruyn et al. (2015) compared the MyotonPro's stiffness parameters to vertical stiffness of the lower limb, measured with a unilateral hopping test, and free oscillation of the calf. Their results showed that the MyotonPro parameters correlated to many performance assessments, such as the eccentric isometric ratio of the quadriceps and hamstring, squat jump or counter-lateral hop test. They found convergent results between the leg's vertical stiffness and the MyotonPro's stiffness parameters.

Based on our results, the MyotonPro revealed itself to be a suitable device for performing *in vivo* measurements of myofascial tissue's viscoelastic properties. Unlike other, more accurate devices such as magnetic resonance elastography (Chakouch et al., 2014), ultrasound shear wave elastography (Gennisson et al., 2013) or an EOD (Detrembleur and Plaghki, 2000; Nguyen et al., 2020), the MyotonPro is by far more portable, affordable, and easy to use, even for a novice operator (Aird et al., 2012; Mullix et al., 2012; Agyapong-Badu et al., 2016). The stiffness measurement collected in passive conditions with the MyotonPro appears to be related to dynamic muscle performances (Pruyn et al., 2015) as well as passive elastic and viscous joint stiffness in the wrist.

### Clinical Implications

Our study confirmed some underlying questions that were not completely answered in regard to MyotonPro assessment. The stiffness parameter is mostly related to passive elastic stiffness. In the neurological field, several authors have studied the usefulness of the MyotonPro in the quantification of spasticity in stroke patients (Fröhlich-Zwahlen et al., 2014; Lo et al., 2017), paratonia patients (Drenth et al., 2018) or patients with spinal cord injury disorders (Ko et al., 2018). In musculoskeletal pathologies, the MyotonPro technology had already been used in myofascial disorders (Jiménez-Sánchez et al., 2018), anterior cruciate ligament rehabilitation (Ortega-Cebrian et al., 2016) and chronic low back pain follow-up (Hu et al., 2018). Based on our results, future studies should focus their attention mostly on the stiffness parameters.

Quantifying and following passive musculoarticular stiffness in myofascial tissues and joints is a growing field of interest among biomechanics specialists, coaches, researchers and clinicians with a view to better understanding movement and its intrinsic properties. This could enhance the validation of therapeutic approaches or sport performance issues (Blackburn et al., 2004; Maquirriain et al., 2012; Pruyn et al., 2014).

### Limitations

This study presented several limitations. Firstly, the stiffness MyotonPro parameters could be confirm in a silicon polymer but a perfect comparison with myofascial tissues should be taken with caution. Secondly, despite showing excellent relative and absolute reliability in the literature, the MyotonPro showed some variability depending on the muscle tested (Agyapong-Badu et al., 2013; Mooney et al., 2015; Feng et al., 2018). Furthermore, the sample population was relatively young and could influence the results (Nguyen et al., 2020). Therefore, we cannot generalize the results to the general population. Thirdly, we used a method of standardization with bone anatomical references rather than manual palpation. We thus tried to increase the reproducibility of measurements; however, we lost the ability to identify anatomical divergences in the tested tissue. Therefore, we could not specify the nature of the tissue targeted by the MyotonPro's probe. Also, we had to normalize the EOD and MyotonPro value in order to perform the Passing & Bablok analysis. Fourthly, we did not assess muscle activity during the myotonometry measurement. This could provide imprecision as whether or not the muscle was activated. Indeed, this could impair the passive stiffness assessment, providing us with unreliable data. However, we performed this assessment in clinical condition and clear instructions to the patient to limit any active muscle contraction. If needed, a pre-trial was performed. Finally, a major limitation was that EOD mobilized the whole joint, measuring overall stiffness while the MyotonPro could only provide localized stiffness measurement, involving specific tissue. Therefore, a high correlation will probably never occur.

## Conclusion

The stiffness and decrement parameters measured with the MyotonPro technology are valid and demonstrated accuracy in identifying materials with different elastic stiffness. Furthermore, the MyotonPro frequency, stiffness relaxation time and creep of the forearm showed small but significant correlations with passive elastic and viscous stiffness of the wrist. More particularly, the proximal and distal anterior myofascial tissues of the forearm showed a small relationship with the elastic stiffness of the wrist while the distal tissue of the posterior side of the forearm demonstrated a relationship with the viscous stiffness of the wrist.

## Data Availability Statement

The raw data supporting the conclusions of this article will be made available by the authors, without undue reservation.

## Ethics Statement

The studies involving human participants were reviewed and approved by Comité d'Ethique Hospitalo-Facultaire. The patients/participants provided their written informed consent to participate in this study.

## Author Contributions

AN: conceptualization, methodology, validation, formal analysis, investigation, writing—original draft, and visualization. CD: conceptualization, methodology, formal analysis, writing—review and editing, and project administration. PF: resources and writing—review and editing. CS: investigation and writing—review and editing. PM: conceptualization, methodology, investigation, writing—review and editing, and supervision. All authors contributed to the article and approved the submitted version.

## Conflict of Interest

The authors declare that the research was conducted in the absence of any commercial or financial relationships that could be construed as a potential conflict of interest.

## Publisher's Note

All claims expressed in this article are solely those of the authors and do not necessarily represent those of their affiliated organizations, or those of the publisher, the editors and the reviewers. Any product that may be evaluated in this article, or claim that may be made by its manufacturer, is not guaranteed or endorsed by the publisher.
